# Supplementary material for: MiR-21 Is Induced by Hypoxia and Down-Regulates RHOB in Prostate Cancer
Source: Cancers (Basel). 2023 Feb 17;15(4):1291. doi: 10.3390/cancers15041291 (PMC9954526; doi:10.3390/cancers15041291)
Supplement: Supplementary file 1 [file cancers-15-01291-s001.zip › cancers-2134049-supplementary.pdf]

SUPPLEMENTARY FIGURES, Angel et al, 2023; MiR-21 is induced by hypoxia and down-regulates *RHOB* in prostate cancer.

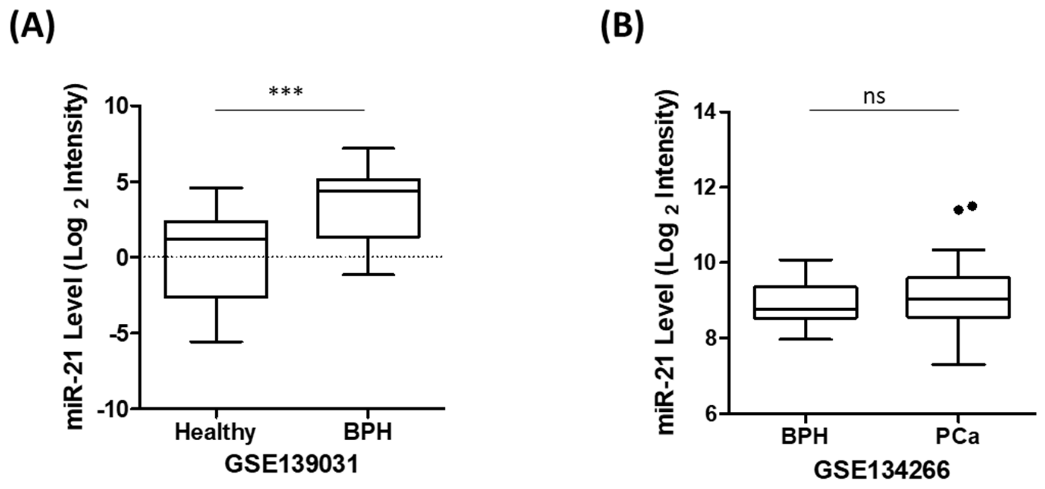

Figure S1. Circulating levels of miR-21 in Benign Prostatic Hyperplasia
(A) miR-21 is significantly elevated in the serum of Benign Prostatic Hyperplasia (BPH) patients (n = 39) compared to healthy, non-cancer control patients (n = 157). Data from GEO dataset GSE139031. (B) no significant difference in miR-21 serum levels between BPH patients (n = 9) and prostate cancer (PCa) patients (n = 29). Data from GEO dataset GSE134266. (Both Welch's t-test, ***$p < 0.001$).

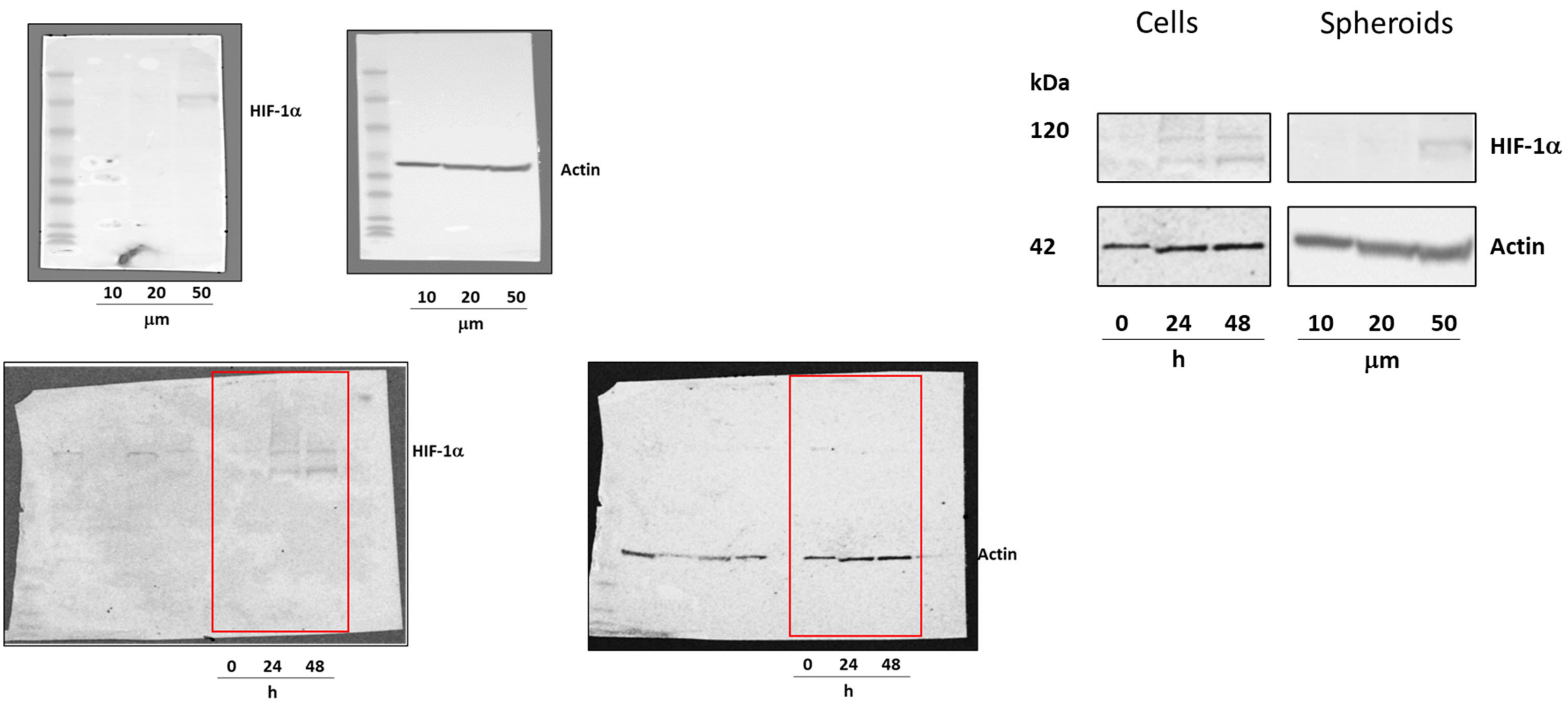

Figure S2. HIF-1α protein expression. Protein from LNCaP monolayer cells and LNCaP spheroids was analysed by Western blot, using antibodies for HIF-1α and α-Actin (both Sigma). Representative cropped western blots (top right), with uncropped and unedited versions of Westerns blots also shown.

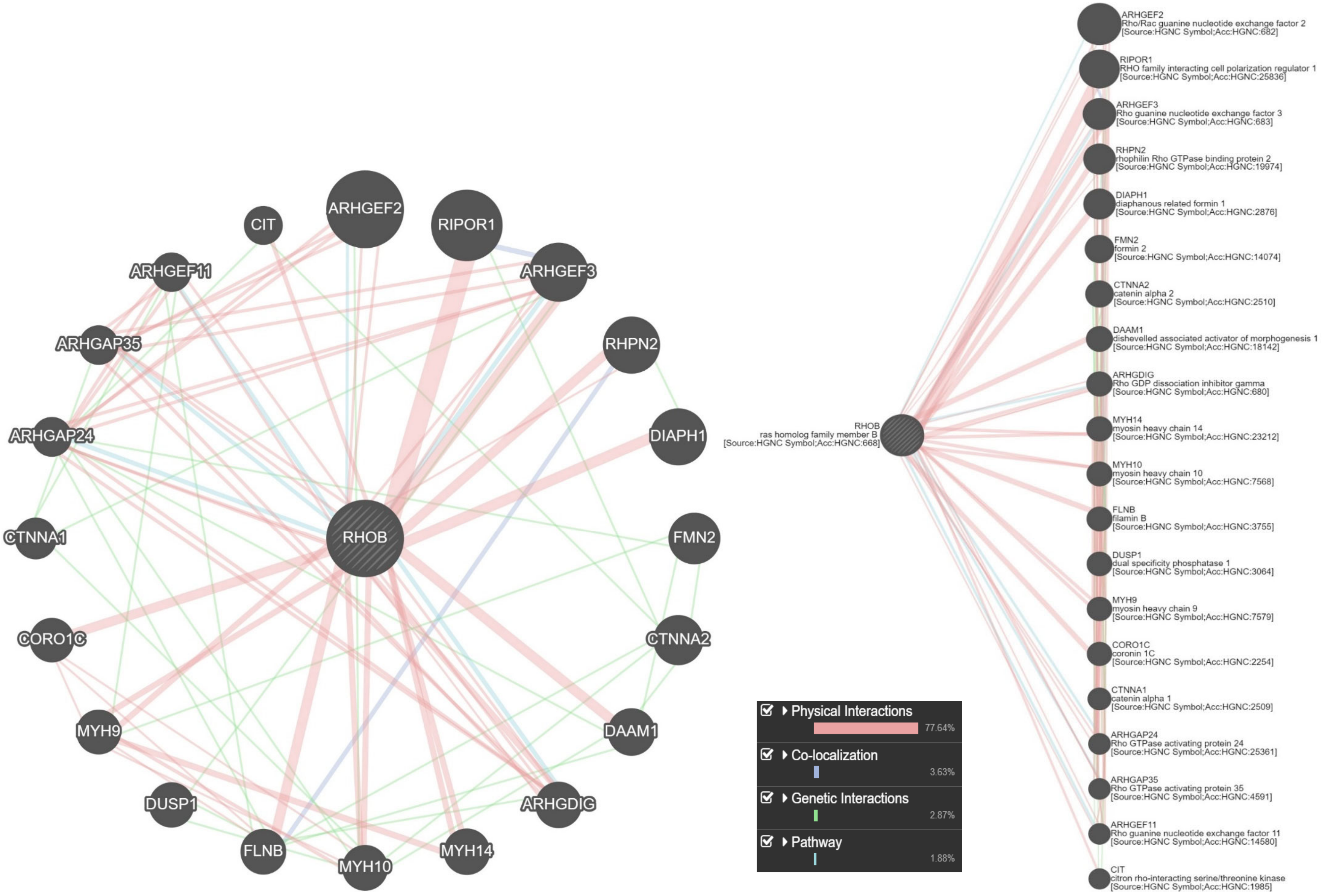

Figure S3. Network analysis of *RHOB* interactions
Visualization by GeneMANIA

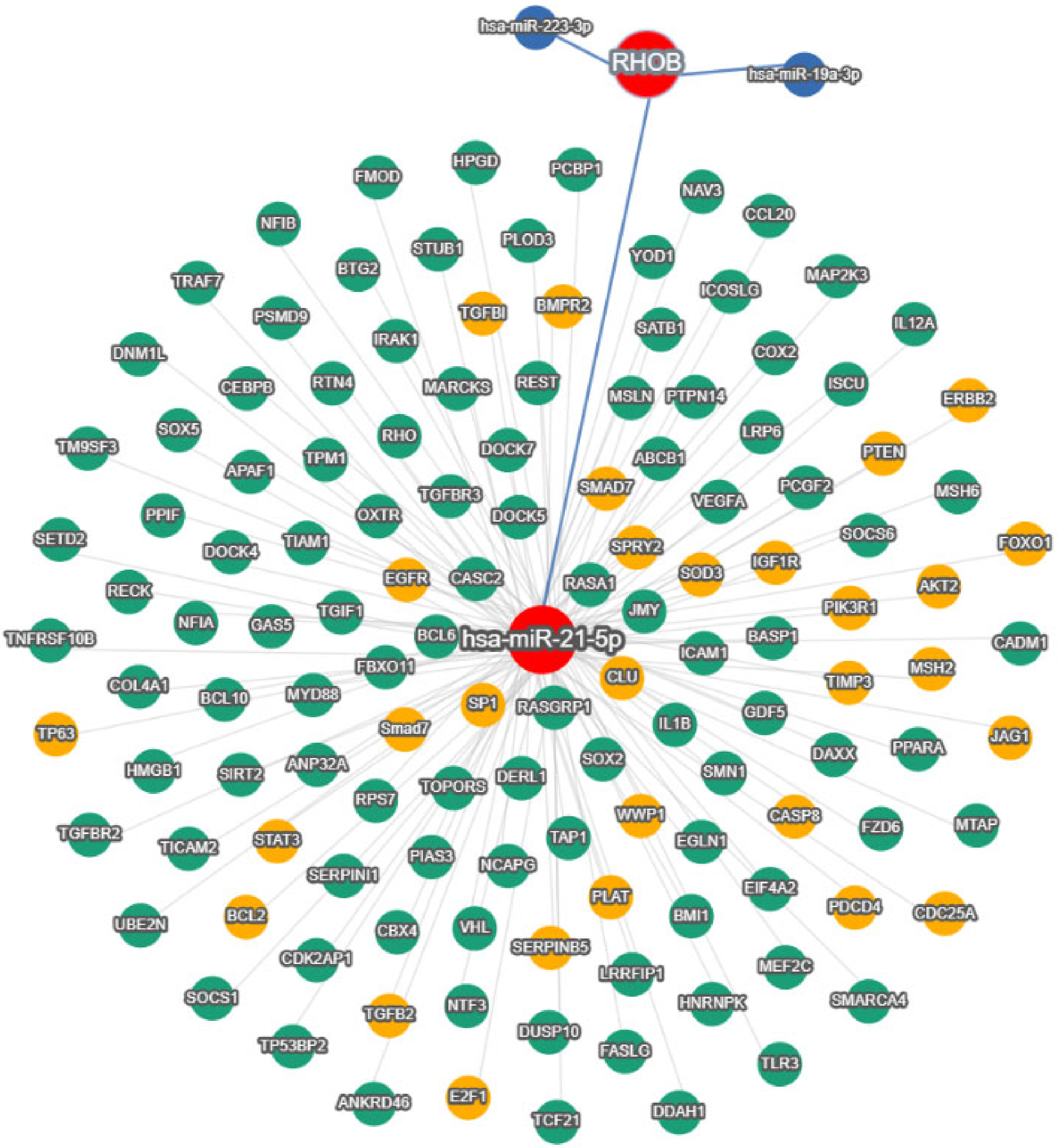

Figure S4. Network analysis of miR-21-5p and *RHOB* interactions

At the top, the two miRNAs in blue (miR-223-3p and miR-19a-3p) are those that have also been shown to target *RHOB* by strong experimental evidence (Luciferase assay, western blot, qRT-PCR). Similarly, the genes shown are those for which strong experimental evidence exists that they are targeted by miR-21-5p. Of these, the genes highlighted in yellow are those that are consistently associated with prostate cancer. Visualization by miRTargetLink 2.0.

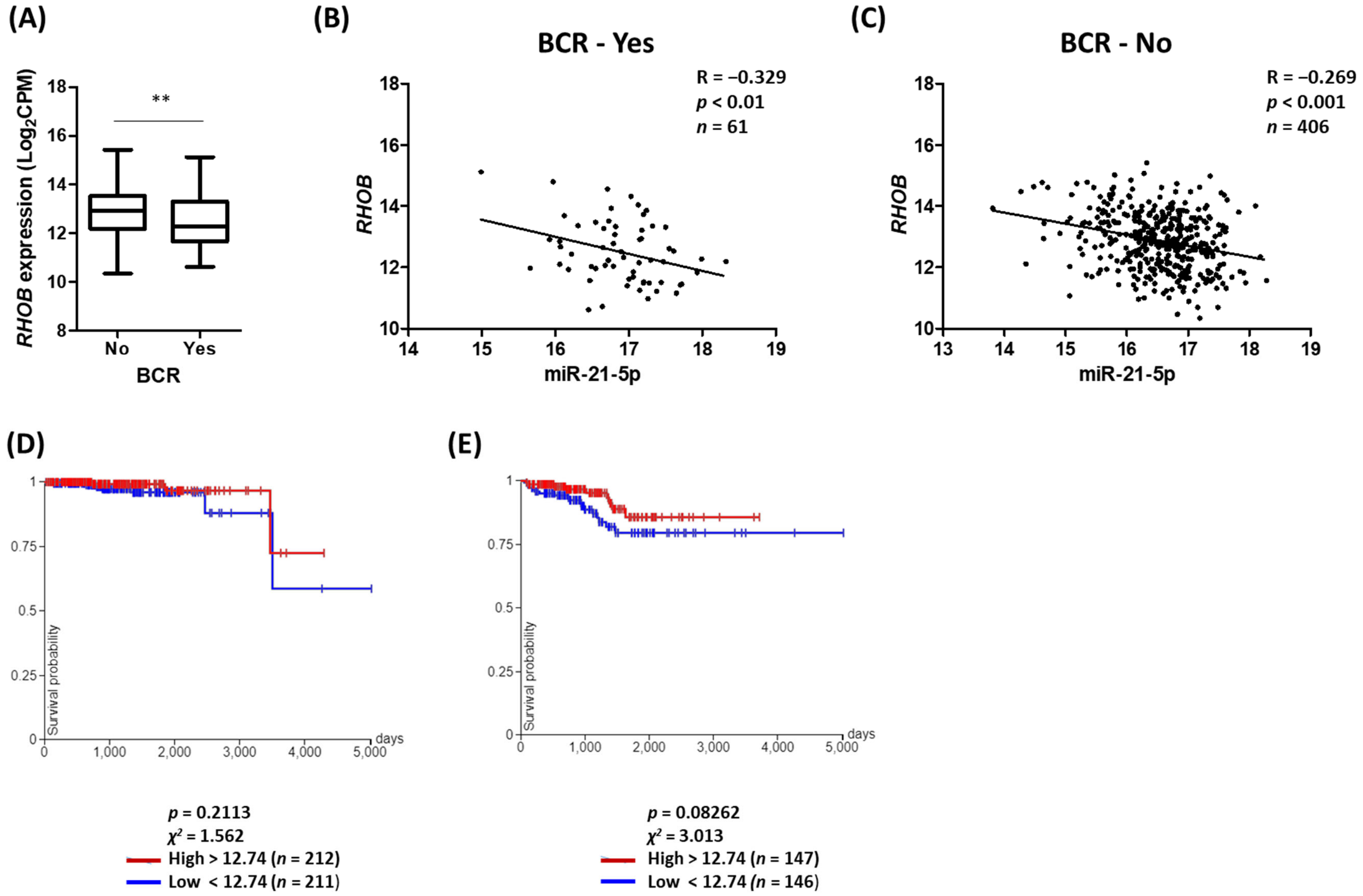

Figure S5. Potential of *RHOB* as a biomarker of prostate cancer

(A) Biochemical recurrence is associated with significantly low levels of *RHOB* (*n*, no recurrence = 406, recurrence = 61). Welsh's *t*-test $^{**}p < 0.01$. *RHOB* and miR-21-5p expression are significantly inversely correlated in patients (B) with BCR and (C) with no BCR. KM survival curves show *RHOB* levels are not significantly associated with (D) Overall survival or (E) Disease free interval. Log-rank (Mantel-Cox) test. Analysis was performed using the UCSC Xena based on prostate cancer cohort in TCGA database.

Table S1. Random forest model based on 500 decision trees showing how the combination of the three variables produces a model that can predict patient biochemical recurrence with accuracy >90%, sensitivity >50%, specificity 100%, $p < 0.001$.

**Random Forest Model (500 iterations)**<br>Variables: Gleason grade, miR-210 & miR-21<br>Outcome: Biochemical recurrence		
Accuracy	:	0.9097
95% CI	:	(0.8697, 0.9407)
No Information Rate	:	0.8159
p-value [Acc>NIR]	:	0.00001
Kappa	:	0.6292
McNemars's test *p*-value	:	0.000002
Sensitivity	:	0.5098
Specificity	:	1
Pos. Pred. Value	:	1
Neg. Pred. Value	:	0.9004
Prevalence	:	0.18412
Detection Rate	:	0.09386
Detection Prevalence	:	0.09386
Balanced Accuracy	:	0.7549

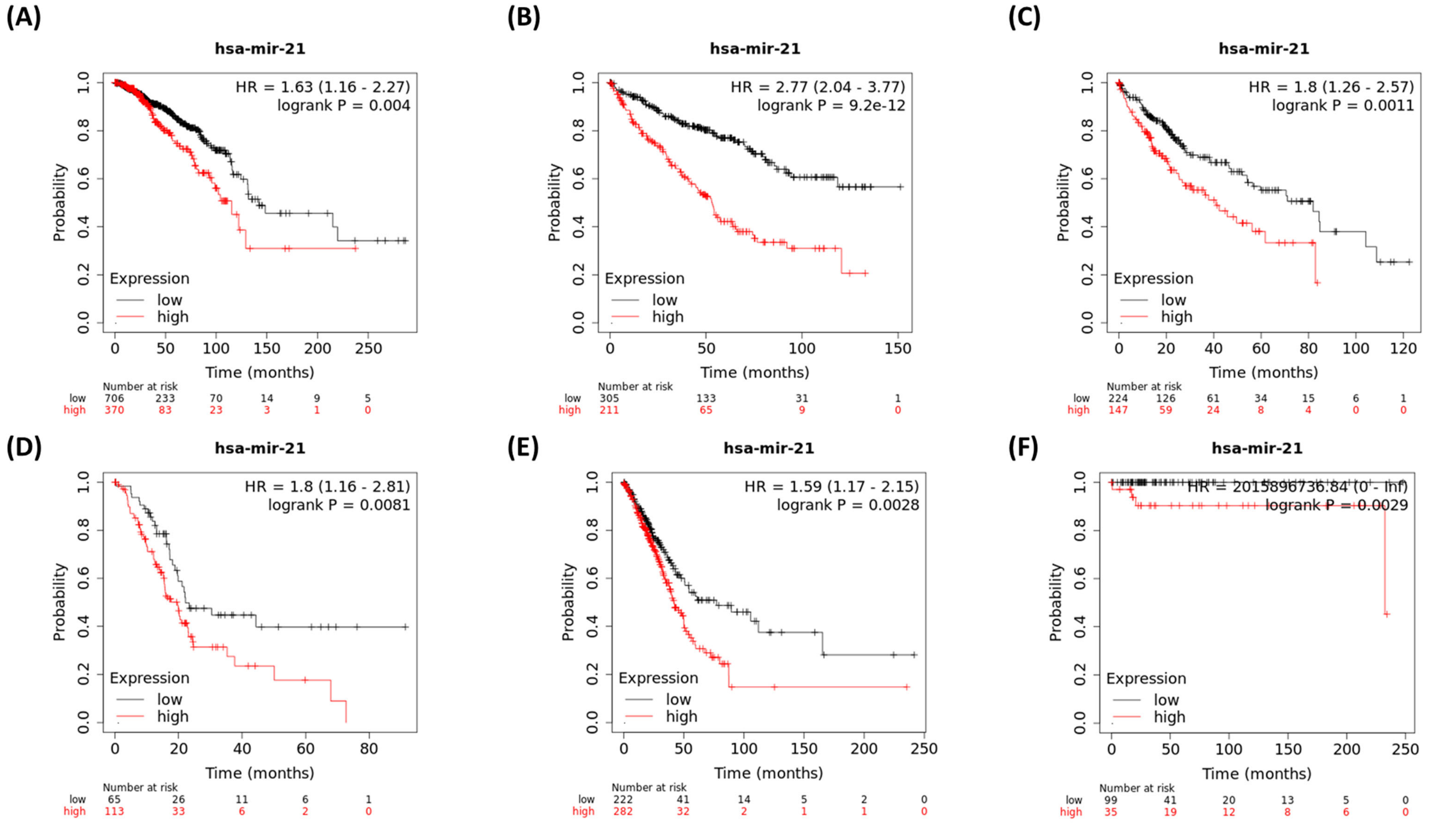

Figure S6. miR-21 as a biomarker of poor survival in other cancers

Kaplan-Meier plots show high miR-21 expression in tumour tissue is associated with significantly poorer survival in (A) breast cancer (B) kidney renal clear cell carcinoma (C) liver hepatocellular carcinoma (D) pancreatic ductal adenocarcinoma (E) lung adenocarcinoma and (F) testicular germ cell tumour. Visualisation by KM Plotter.

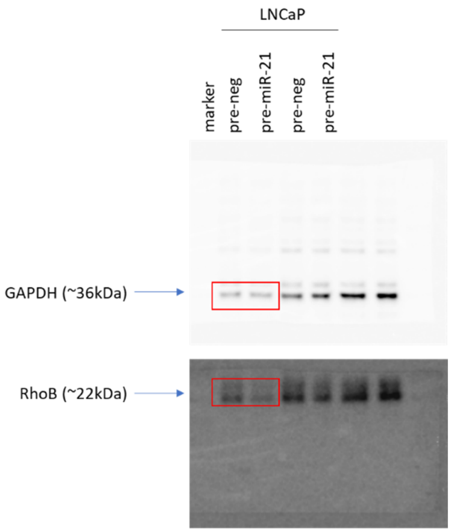

Figure S7. RhoB protein expression.
Protein from LNCaP cells, transfected with miR-21 'pre-miR-21' and non-targeting control 'pre-neg' was analysed by Western blot. Uncropped versions of the blots shown in Figure 3B.
